# Education as the Great Equalizer? Racial and Ethnic Differences in the Effect of Education on Cognitive Impairment in Later Life

**DOI:** 10.3390/geriatrics4030051

**Published:** 2019-09-05

**Authors:** Kyler J. Sherman-Wilkins, Amy D. Thierry

**Affiliations:** 1 Department of Sociology and Anthropology, Missouri State University, Springfield, MO 65897, USA; 2 Department of Public Health Sciences, Xavier University of Louisiana, New Orleans, LA 70125, USA; athierry@xula.edu

**Keywords:** education, racial/ethnic differences, cognitive functioning, cognitive impairment

## Abstract

Though evidence suggests that the prevalence of cognitive impairment has declined, there still exists a disproportionate burden of ill cognitive health for people of color. In this paper, we test two alternative mechanisms to explain the interactive effect of education and race/ethnicity on cognitive impairment risk: the minority poverty and diminishing returns hypotheses. Drawing on data from the 2012 wave of the Health and Retirement Study (HRS) (*n =* 8093), we estimate logistic regression models to determine differential effects of education on cognitive impairment. We find that non-Hispanic black and Mexican American older adults have higher odds of being cognitively impaired compared to whites, though the ethnic difference (whites vs. Mexican Americans) is mediated by education. Further, we find that while high levels of education are protective against cognitive impairment at older ages, it is more protective for non-Hispanic blacks than for whites and more protective for whites than Mexican Americans. Lastly, we find that racial/ethnic disparities are widest at lower levels of education, consistent with the minority poverty hypothesis. We conclude that the results herein highlight the importance of attending to how factors that are protective for cognitive functioning (e.g., education) may operate differently across racial and ethnic groups.

## 1. Introduction

In January of 2011, President Barack Obama signed the National Alzheimer’s Project Act into law, cementing the federal government’s commitment to addressing the high prevalence of dementia and cognitive impairment in the United States. Though recent evidence suggests that the prevalence of severe cognitive impairment has declined significantly between 2000–2012 [1], there still exists a disproportionate burden of ill cognitive health for people of color. Indeed, numerous studies have found that relative to whites, blacks and Hispanics suffer from poorer cognitive functioning across the lifespan [2,3,4]. A significant portion of the racial/ethnic difference in cognitive functioning in late life can be attributed to differences in education, income, and adulthood health behaviors [4,5].

Given evidence that social conditions matter for health outcomes [6], investigators have increased focus on the social determinants of cognitive impairment. Level of education has consistently been shown to be a robust predictor of cognitive functioning in later life [7]. Studies typically include indicators of education as a mediator with the goal of ascertaining whether racial/ethnic differences in education explain racial/ethnic differences in cognitive functioning [2,4,5,8,9]. However, a growing number of studies have examined whether the effect of education on cognitive functioning is the same across race/ethnicity in older adults [10,11,12]. Though our understanding of how education may differentially affect cognitive health across race and ethnicity has increased, a number of questions remain unanswered. First, existing research has examined cognitive functioning [10,11,12]. Cognitive impairment is distinct from cognitive functioning [13] and has, to our knowledge, not been thoroughly investigated in the extant literature on racial differences in the returns of education. Second, there is a need for research using a nationally representative sample of older adults, which will allow for generalizations to be made. Third, while black-white differences have been explored, there is a shortage of studies that consider how the effects of education vary across Hispanic ethnicity. Lastly, the research on differential effects of education on cognition has largely been separate from research testing two potential mechanisms for conditional effects of education across race, namely the minority stress and diminishing returns hypotheses.

In this paper, we investigate whether the effects of education on cognitive impairment in later life are different across race and ethnicity—with a particular focus on non-Hispanic whites, non-Hispanic blacks, and Mexican Americans. Drawing on data from the 2012 wave of the nationally representative Health and Retirement Study (HRS), we estimate a series of nested logistic regression models to examine differential effects of years of schooling on cognitive impairment. Specifically, we test two competing hypotheses for how the effects of education vary across race and ethnicity: (a) the minority poverty hypothesis and (b) the diminishing returns hypothesis.

## 2. Background

That there is a direct relationship between high socioeconomic status (SES) and good health has become somewhat of an axiom in the literature on the social stratification of health [9]. Mechanisms underlying the association between SES and health largely include relative access to various forms of capital and resources which individuals can then mobilize to stave off adverse health [14,15]. For a more concrete example, individuals with more income are likely to afford healthier foods or be able to afford better healthcare treatment than those of lower income.

SES is essentially a proxy of social class and is measured as a combination of education, income, wealth, and occupational status/prestige, but a number of studies have focused explicitly on education. Indeed, education seems to both directly and indirectly affect the other indicators of SES [15]. In the context of cognitive functioning, explicit focus on education is also warranted because of its consistent and robust association with cognition [7,10,11,12]. Given the strong relationship between education and cognition as well as the strong evidence for racial/ethnic disparities in cognitive impairment [2,10,11,12], it is worth investigating whether differences in education drive said disparities in cognitive aging. Does education serve to mediate the relationship between race/ethnicity and cognitive impairment, or rather does education operate differently across race/ethnicity?

### 2.1. Racial/Ethnic Differences in Effects of Education on Health and Cognition

Previous research has provided evidence for differential benefits of education on health. For instance, Farmer and Ferraro find that blacks do not receive as great a benefit from education as whites with regards to self-rated health [16]. Similarly, Shuey and Willson’s 2008 study of black-white disparities in life-course health trajectories provided support for education being less protective for blacks than it is for whites when predicting health trajectories [17].

Turning attention specifically to investigations of differential benefits for cognitive functioning, there is some evidence that the relationship between education and late-life cognitive functioning varies across race. Drawing on a community-based sample of older blacks and whites in Chicago, Barnes and colleagues found that high levels of education were more strongly associated with better cognitive performance in blacks than it was for whites [10]. Expanding on this work, Sisco and her team analyzed longitudinal data from community dwelling older adults in the Washington Heights-Inwood area and found that education quality was predictive of cognitive functioning among blacks but not whites [11]. Conversely, Wilson and colleagues found no evidence that the relationship between education and cognitive function is different for blacks and whites [12]. Differences across studies are likely due to the domains of cognition measured and/or the nature of the analytic sample.

### 2.2. Alternative Mechanisms for Differential Effects of Education on Cognitive Impairment

Though there is growing evidence for differential effects of education on cognitive functioning, there is not yet consensus on whether education operates more strongly for racial minorities or their white peers. In their examination of the conditionality of SES indicators including education in determining racial disparities in self-rated health, Farmer and Ferraro test two competing hypotheses: the minority poverty hypothesis and the diminishing returns hypothesis [16]. The minority poverty hypothesis predicts that disparities in health are widest at lower ends of SES, but then decline as one moves up the SES ladder. This hypothesis is rooted in the literature that finds that blacks face unique threats and challenges when living in poverty [18]. Said unique threats and challenges are a result of experiencing stressors at the intersection of race and class [19,20]. In the context of this analysis, the minority poverty hypothesis predicts that racial disparities in cognitive aging are largest at lower levels of education.

Conversely, the diminishing returns hypothesis predicts that, while health disparities across social categories exist at all levels of SES, the disparities are greatest at high levels of SES and reflect a process where racial/ethnic minorities are not able to translate their human capital into resources to benefit their health. Previous work has documented that compared to whites, blacks do not benefit as much from the resources that high education grants them access to [21,22], and, consequently, they are not afforded the same health benefits of high education. In the context of the present research, the diminishing returns hypothesis predicts that racial disparities in cognitive impairment are largest at high levels of education. In sum, both the minority poverty and diminishing returns hypotheses posit that the effects of education on health are conditional on race, but articulate different effects of low and high education across race/ethnicity.

Research is inconclusive with regards to which hypothesis is more correct when predicting health outcomes, with some evidence supporting the minority poverty hypothesis [23,24] and other research providing support for diminishing returns [16]. Inconsistencies in results may be due to the specific health outcomes assessed. While these two hypotheses have not been explicitly tested in the context of cognitive impairment, there seems to be evidence that the returns of higher education are stronger for blacks than whites when looking at indicators of global cognitive functioning [10].

### 2.3. The Importance of Investigating Cognitive Aging Experience of Older Mexicans

Previous research on cognitive impairment using Mexican American samples has shown that while certain segments of this population have increased prevalence of cognitive impairment, education does not operate consistently in predicting cognitive impairment [25]. Moreover, research suggests that cognitive impairment may be linked to physical health and mortality for Mexican Americans [26]. Given concerns over trajectories of cognition and health among Mexican Americans, limited comparisons of Mexican Americans to non-Hispanic blacks and whites hinders understanding of how risk factors such as education contribute to cognitive impairment across racial/ethnic groups. Therefore, including Mexican Americans in research with non-Hispanic blacks and whites will allow us to identify unique processes underlying cognitive impairment for these segments of the U.S. population.

In line with previous research regarding the stratification of cognitive health across racial and ethnic lines, we formulate the following hypothesis.

**Hypothesis** **1 (H1):** *Relative to non-Hispanic whites, non-Hispanic blacks and Mexican Americans are at greater risk of cognitive impairment.*

As previously mentioned, it has been shown that differences in years of education explains a portion of the racial/ethnic gap in cognitive functioning. As such, we articulate our second hypothesis.

**Hypothesis** **2 (H2):** *Years of education will partially mediate the racial/ethnic difference in cognitive impairment.*

Our central argument is that education’s impact on cognitive impairment varies across race/ethnicity, specifically that the racial/ethnic disparity in cognitive impairment would be largest at higher levels of education and smallest at lower levels of education. Therefore, we make the following hypothesis.

**Hypothesis** **3 (H3):** *Racial/ethnic disparities in cognitive impairment are greatest at the highest levels of education reflecting diminishing returns of the protective effect of education for blacks and Mexican Americans.*

Whereas our third hypothesis is consistent with the diminishing returns hypothesis, it is also possible that education affects whites and racial/ethnic minorities differently at the low end. In other words, we would expect racial/ethnic differences in cognitive impairment to be greatest at lower levels of education. Thus, inspired by the minority poverty hypothesis we formulate our fourth hypothesis.

**Hypothesis** **4 (H4):** *Racial/ethnic disparities in cognitive impairment are greatest at the lowest levels of education reflecting differential susceptibility to poor education for blacks and Mexican Americans.*

In testing the aforementioned hypotheses, this paper builds on the existing literature in three key ways. First, we test the idea of differential effects of education on cognitive impairment as opposed to cognitive functioning, an outcome that, to our knowledge, has not been considered before as it relates to the minority stress and diminishing returns hypotheses. Second, while previous studies have restricted their analyses to non-Hispanic blacks and whites, we expand on existing work and include Hispanics, specifically Mexican Americans. In so doing, we examine the largest Hispanic ethnic group in the U.S. and thereby further increase understanding of the aging experience among an increasingly important demographic. Last, we draw on a nationally representative dataset allowing us to generalize our findings to the larger population of older adults.

## 3. Materials and Methods

### 3.1. Sample

Data for this study were drawn from the 2012 nationally representative, longitudinal Health and Retirement Study (HRS). The HRS is sponsored by the National Institute on Aging (grant number NIA U01AG009740) and is conducted by the University of Michigan. Initiated in 1992, the HRS and its sister survey Study of Asset and Health Dynamics Among the Oldest Old (AHEAD) were both conducted separately and biennially, before being integrated in 1998. The HRS collects measures on the health, employment, and familial conditions of non-institutionalized older adults aged 50+ in the United States via in-person interview or by telephone.

One feature of the HRS is its inclusion of various cohort samples to better represent the range of birth cohorts from the 1900s to later in the 21st century. In total, the HRS consists of six cohorts: the AHEAD cohort (born before 1923), the HRS cohort (1931–1941), the Children of the Great Depression (CODA) cohort (1924–1936), the War Babies cohort (1942–1947), the Early Baby Boomers (1948–1953), and the Mid Baby Boomers (1954–1959). While the AHEAD, HRS, CODA, and War Babies were all included in the integrated 1998 assessment, the Early and Mid-Baby Boomers were not added until 2004 and 2010, respectively. The 2012 wave includes all the surveyed cohorts to date.

The HRS employs a multi-stage national area probability sample design. During the first stage, U.S. Metropolitan Statistical Areas (MSAs) and non-MSA counties were selected using probability proportionate to size (PPS). Second, area segments were selected from the sampled primary sampling units (PSUs). Third, once a complete enumeration of all the housing units within the boundaries of the identified area segments is completed, housing units are selected systematically. The fourth and last stage consists of the selection of the specific household financial unit. Additionally, the HRS includes an oversampling of blacks and Hispanics as well as an oversample of Floridians to ensure adequate numbers of members of these groups.

The data source is highly appropriate for the present analyses for several reasons. First, the coverage of older Americans across a wide age range gives us the ability to assess cognitive impairment across older adults, broadly defined. Second, the oversampling of blacks and Mexican Americans ensures that we will have adequate statistical power to make meaningful comparisons across race and ethnicity. Third, in addition to containing an extensive battery of cognitive tests that tap into key domains of cognition, the HRS also includes a number of sociodemographic, health, and health behavior variables that allow for a thorough examination of cognitive impairment and the various factors associated with said impairment. To extract the data, we relied on the RAND HRS Data file as well as “fat files” found on the HRS website (http://hrsonline.isr.umich.edu/).

Our analytic sample consists of respondents aged 65+ in 2012 (*n =* 8903). We limit the analyses to blacks, whites, and Mexican Americans. As discussed earlier, Mexican American older adults experience unique challenges and their growing share of the U.S. population warrants further attention. To account for the oversampling of blacks and Mexican Americans, we used sampling weights to adjust point estimates and standard errors.

### 3.2. Measures

Cognitive impairment, dichotomized to reflect those who are afflicted with impairment (1) and those who are unimpaired (0), served as our dependent variable. To construct the dependent variable, we relied on the modified Telephone Interview for Cognitive Status (TICS) which contains several tasks including object naming, serial subtraction, and both immediate and delayed word recall. The scores from the modified TICS range from 0 to 35, with higher scores denoting better cognitive functioning. In accordance with previous research [4,27,28], we used a cutoff of 9, with those at or below 9 characterized as cognitively impaired, while those above 9 were characterized as unimpaired.

Our focal independent variables included years of education, race, and ethnicity. Years of education (0–17) is treated as a continuous measure denoting the respondent’s educational attainment. Race is a binary variable coded ‘1′ for non-Hispanic blacks, while ethnicity is a binary variable coded ‘1′ for Mexican Americans, with non-Hispanic whites serving as the reference group. To test the hypothesis that the returns of education vary across race and ethnicity, we also constructed interaction terms (race x education; ethnicity x education).

We also included a number of covariates that have known associations with cognitive health. Number of health conditions was treated as a continuous variable and represented the number of self-reported diagnosed diseases (arthritis, cancer, diabetes, heart disease, hypertension, lung disease, and/or stroke. Psychiatric diagnosis was a binary variable based on self-report of ever being diagnosed with an emotional, nervous, or psychiatric problem (0 = no, 1 = yes). We also used the CES-D cut-off of ≥3 symptoms as indicating depression (0 = no, 1 = yes) [29]. Obesity was treated as a binary indicator that indicated obese individuals with BMI ≥ 30 (0 = not obese, 1 = obese). These measures tap into the overall health of the respondent.

To capture health behaviors, we included indicators for exercise, smoking, and alcohol use. To capture exercise intensity we combined two measures of frequency of moderate and vigorous physical activity. We categorized individuals as hardly ever or never (reference), sometimes (once a week or one to three times a month), or frequently (once a week) engaging in either moderate or vigorous physical activity. Smoking status was constructed using responses to inquiries into whether a respondent never smoked, were former smokers, or current smokers. We constructed alcohol use by examining two questions asked of respondents: whether respondents ever drank an alcoholic beverage, and the number of alcoholic drinks consumed daily. Based on these responses, we generated three categories: those who never drank (coded ‘0′), current moderate drinkers who reported drinking 1‒2 drinks on days when drinking alcohol (coded ‘1′), and current heavy drinkers who reported drinking ≥3 drinks per day when drinking alcohol (coded ‘3′). We relied on the 2015–2020 Dietary Guidelines for Americans, published by the U.S. Department of Health and Human Services and the U.S. Department of Agriculture. Moderate drinking is defined as 1 drink per day for women and up to 2 drinks per day for men.

Sociodemographic controls include age (continuous, ranging from 65–102) and married/partnered (0 = no, 1 = yes) and sex (0 = male, 1 = female). We also included measures of household income and wealth. Due to skewness of income and wealth variables, both were logged transformed. For both income and wealth (calculated as assets minus debts), a constant of $1 was added to each value before taking the log of the absolute value of each. This ensured that individuals reporting $0 in income or wealth were not lost during the log transformation. Then, for those individuals who originally had negative values of wealth, their log transformed values were multiplied by −1. This method is consistent with previous research using income and wealth data from HRS [30].

### 3.3. Analytic Strategy

**Figure 1:**
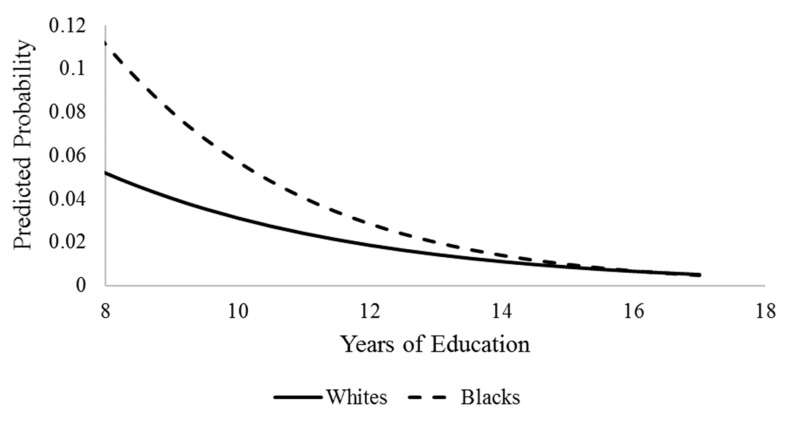
Race by Education Interaction Predicting Cognitive Impairment, HRS 2012.

**Figure 2:**
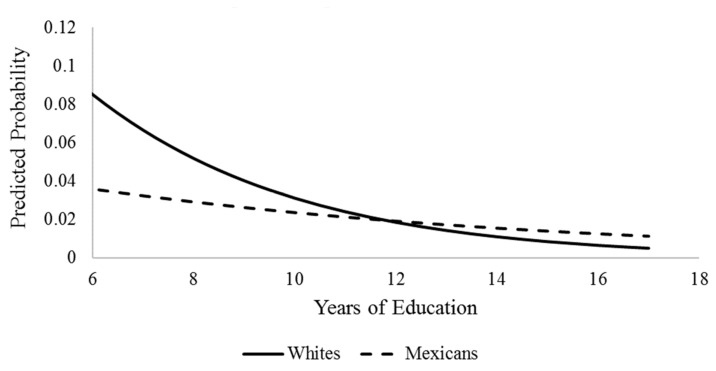
Ethnicity by Education Interaction Predicting Cognitive Impairment, HRS 2012.

We began by generating weighted descriptive statistics for the analytic sample. Table 1 presents said statistics, stratified by race and ethnicity. Next, to assess our research hypotheses, we estimated a series of nested logistic regression models. The binary nature of our dependent variable made the use of logistic regression more appropriate than ordinary least squares regression. Table 2 presents parameter estimates from logistic regression models. Model I examined the direct effect of race and ethnicity on cognitive impairment controlling for sociodemographic and health characteristics. Next, Model II included years of education into the model and tested for the mediation effect of education on racial/ethnic disparities in cognitive impairment. Collectively, Models I and II provide a test of H1 and H2, respectively. Lastly, we tested race/ethnicity x education interaction terms to assess whether the effects of education are different across race/ethnicity. Point estimates are presented in Model III. We generated graphs for all statistically significant interaction terms. Figure 1 and Figure 2 display visualizations of said interactions. Visualizations of the interactions allowed us to better interpret the conditional effects of race/ethnicity and thus test both H3 and H4.

As with all surveys, the HRS is not without missing values. To address missing data, we began by examining patterns of missing data across independent variables and controls. The extent of missing data was low, with no variables having more than 1% missing. We relied on the Rand Corporation’s income, wealth, and cognition imputation. For all other analyses, we used listwise deletion. All analyses were conducted using Stata 15, StataCorp LLC (College Station, TX, USA).

## 4. Results

### 4.1. Descriptive Statistics

Table 1 presents descriptive statistics for all measures. We show proportions for categorical variables and mean values for continuous variables for the entire sample as well make comparisons across race/ethnicity and gender using the adjusted Wald’s test. Blacks and Mexican Americans have lower TICS scores compared to whites, with black men having the largest proportion of adults meeting the ≤9 cutoff at 6.74%. Compared to white men and women, blacks and Mexican Americans in the sample are of lower SES. For instance, Mexican Americans report the fewest years of education (8.93 for men and 7.93 for women). Black and Mexican American women report less income compared to both white men as well as their racial/ethnic male counterparts. Less wealth compared to whites is also reported by blacks and Mexican Americans, with black women having less wealth than black men. Differences in marital/partnered status are indicated, with women of all racial/ethnic groups being less likely to be married/partnered than men.

Racial/ethnic and gender differences are also noted for behavioral risk factors. Black women report a higher prevalence of obesity than both white and black men (44.33% compared to 28.82% and 31.71% respectively). White and black women are more likely to report never participating in moderate/vigorous physical activity compared to their male counterparts. Black and Mexican American men compared to women are more likely to be current or former smokers, while men across all racial/ethnic groups are more likely than women to be moderate or heavy drinkers.

For physical health, white and black women report more health conditions compared to men. Racial/ethnic and gender differences are also present for the two measures of mental health. First, women report a greater prevalence of ever being diagnosed with a psychiatric problem than men regardless of race/ethnicity. However, black men report a lower prevalence of ever-diagnosed psychiatric problems compared to white men (9.61% vs. 13.94%). Similar to psychiatric diagnoses, having a CES-D ≥3 as an indication of depression is more common in white and black women than men. Additionally, Mexican American men are more likely than white men to have CES-D scores ≥3.

### 4.2. Multivariate Analyses

Table 2 presents results from the multivariate logistic regression analyses testing our hypotheses. All models control for demographics (age, gender, log income, log wealth, and marital/partnership status), health status (number of health conditions, psychiatric diagnoses, and CES-D score), and behavioral risks (obesity, moderate/vigorous physical activity, smoking status, and alcohol use). First, Model I shows that blacks have a greater likelihood than whites of being cognitively impaired (OR:3.56, CI:2.44–5.19, *p* < 0.001). Mexican Americans also were more likely than whites to fall within the cognitively impaired category (OR:1.96, CI:0.1.04–3.90, *p* < 0.05). Therefore, Model I represents the baseline disparity in cognitive impairment for black and Mexican American older adults compared to whites even after accounting for multiple sociodemographic and health related factors.

Turning to Model II, education altered the relationship between race/ethnicity and odds of cognitive impairment. The main effect being that non-Hispanic blacks fell from 256% higher odds relative to non-Hispanic whites to 166% higher odds. The non-Hispanic black-white difference remained highly significant, but the magnitude of the odds ratio was reduced by a little over a third. This attenuation in the main effect provides some evidence for partial mediation. Whereas there was partial mediation of the race effect for non-Hispanic blacks, education completely mediated the difference between Mexican Americans and non-Hispanic whites. Indeed, the point estimate for the ethnicity effect went from significant to not significant. Moreover, after accounting for education, Mexican Americans had lower odds than non-Hispanic whites of being cognitively impaired. In terms of H2, then, we found mixed evidence. There was slight mediation of education for blacks and complete mediation for Mexican Americans.

### 4.3. Conditionality on Race/Ethnicity

Model III includes point estimates for race/ethnicity by education interaction terms. Figure 1 and Figure 2 provide visual displays of the statistically significant interaction terms identified in our third logistic regression equation. First, Figure 1 displays the statistically significant interaction between race and education. As the figure indicates, non-Hispanic blacks displayed a higher predicted probability of cognitive impairment relative to non-Hispanic whites, particularly at the lowest levels of education, suggesting that lower levels of education are more detrimental to non-Hispanic blacks than for their white peers. The disparity in probability of poor cognitive health began to shrink as education increased. The convergence in the predicted probability of cognitive impairment was found to be driven by the stronger effect of higher education for non-Hispanic blacks relative to their non-Hispanic white peers. The patterns displayed here do not provide support for H3, and instead are more in line with the prediction made by H4.

Figure 2 graphs the detected interaction between Mexican ethnicity and education. While non-Hispanic blacks were found to experience higher predicted probabilities of cognitive impairment than non-Hispanic whites at all levels of education, Mexican Americans had a lower predicted probability of being cognitively impaired than non-Hispanic whites. This pattern played out at all levels of education up until around 12 years. After 12 years of education (equivalent of high school graduate), non-Hispanic whites displayed a lower probability of impairment. The pattern of disparity in cognitive impairment across ethnicity is driven largely by the stronger returns of education for non-Hispanic whites than for Mexican Americans. Indeed, Mexican Americans maintain a low probability of impairment across years of education and experience only a marginal return to their cognitive health. As was the case for Figure 1, the disparity in cognitive impairment between Mexican Americans and whites is widest at lower levels of education. Results herein are contrary to the prediction made in H3, while H4 receives support. It is important to note that caution should be taken when interpreting the results laid out in Figure 2. While the results are significant, issues with sample size prevent us from having full certainty in the estimates. We will discuss this limitation further in the discussion section.

## 5. Discussion

This study examined the association between education and cognitive impairment by race/ethnicity among a nationally representative sample of US adults ≥65 years of age. We tested the hypothesis that non-Hispanic blacks and Mexican Americans would have a greater risk for cognitive impairment compared to non-Hispanic whites. We also assessed if years of education would mediate racial/ethnic differences in cognitive impairment. Furthermore, we aimed to test two hypotheses proposed in the literature to explain how education might operate on health —the minority poverty hypothesis and the diminishing returns hypothesis. We tested whether increasing years of education would provide blacks and Mexican Americans similar protection against cognitive impairment as for whites. Support for the diminishing returns hypothesis would entail blacks and Mexican Americans maintaining greater likelihood of cognitive impairment than whites even at the highest level of education. Conversely, narrowing of the gap in cognitive impairment between blacks or Mexican Americans and whites with increasing years of education would provide evidence for the minority poverty hypothesis.

We find that black and Mexican American older adults have higher odds of being cognitively impaired compared to their white peers. For example, blacks were found to have 256% higher odds of being cognitively impaired compared to whites, while Mexican Americans had 96% higher odds of cognitive impairment than whites. These disparities were present even with the inclusion of various sociodemographic, health status, and behavioral risk measures. Thus, we examined specifically the effect of years of education on these racial/ethnic disparities. We found that years of education fully attenuated the difference in odds of cognitive impairment for Mexican Americans; however, blacks continued to have 166% higher odds of cognitive impairment compared to whites.

Next, our analysis of interaction terms between years of education and race and ethnicity showed that the effects of education on cognitive impairment is shaped by race and ethnicity. First, non-Hispanic blacks significantly benefit from more years of education, such that the cognitive impairment gap between non-Hispanic blacks and whites steadily decreases with increasing years of education and ultimately closes at the highest level of education. Thus, this provides support for the minority poverty hypothesis as disparities are concentrated at lower levels of education while more educational attainment appears to reduce black adults’ likelihood of cognitive impairment. Our findings are consistent with Barnes and colleagues’ work that found that Chicago-based blacks experience greater returns of higher education to cognitive functioning in later life [10].

When comparing the effect of education on cognitive impairment between Mexican Americans and whites, we found an interesting pattern. While we still find that the disparity in cognition is widest at the lower levels of education, we also find that it is non-Hispanic whites that have a higher probability of cognitive impairment then Mexican Americans across education level, a result that is contrary to the minority poverty hypothesis. Further, with increasing years of education, non-Hispanic whites exhibit lowering of the odds of being cognitively impaired while the impact of increasing years of education is minimal for Mexican Americans. Thus, at least when thinking about Mexican American and non-Hispanic white disparities in cognitive impairment, it is the majority and not the minority group that is disadvantaged more by low levels of education. We argue that there are two possibilities for these findings. First, it is possible that these results reflect the so-called Hispanic health paradox, which posits that even in spite of lower SES, Hispanics in the U.S. fair better than their non-Hispanic white counterparts in regard to health [31]. While this phenomenon is consistently shown for various health conditions and mortality, research using cognition as an outcome presents mixed results [32]. A second possibility is that the results are merely an artifact of our modeling choices and inadequate statistical power given the small number of Mexican Americans in the analytic sample coded as having cognitive impairment. We discuss this possibility more in the limitations section.

The findings herein have implications for how we address disparities in cognitive impairment, at least as it relates to education. Based on the results, it would seem that the most effective way to lessen racial/ethnic inequality in cognitive impairment would be to promote education among disadvantaged groups. While our results suggest that promoting education may lessen disparities in cognitive health, it is important to note quality of education and not necessarily years of education may matter more. Indeed, research conducted by Sisco and colleagues shows that early life education quality explains a significant portion of racial disparities in cognitive functioning in later life [11]. Research has also shown that literacy may matter more for racial disparities in cognitive impairment than years of schooling [33]. With this in mind, future research is needed to tease out the mechanisms by which education affects cognitive health across racial and ethnic lines.

### 5.1. Strengths and Limitations

It is important to view these findings within the context of strengths and limitations. One strength of the present study is that it draws on a large, nationally representative sample of racially and ethnically diverse US adults ≥65 years of age as opposed to select, community-based samples. Second, we were able to focus on a specific Hispanic ethnic group, Mexican Americans, as opposed to grouping all Hispanics together and thus obscuring important differences between Hispanics from different cultural backgrounds. Indeed, focusing on Mexican Americans alone allows for generalizability of results for targeted intervention to reduce health disparities in the largest Hispanic group in the U.S. Third, because the HRS provides several sociodemographic and health measures, we were able to control for multiple variables to better identify the role of education beyond other factors important for cognitive impairment. Furthermore, we utilized a validated measure of cognitive functioning that has been employed in several studies of cognitive functioning among older adults in the U.S.

This study is not without some limitations. First, we relied on one wave of the longitudinal HRS and conducted cross-sectional analyses, thus not fully leveraging the rich information contained in the dataset. Therefore, we can only assess the impact of education on cognitive impairment at one point in time. We would like to note, however, that though cross-sectional, participants in the HRS completed their formal education decades before cognition was measured. As such, we can have some confidence in the causal direction between education and cognitive impairment. Nevertheless, future research should assess multiple waves of data to assess how education and other measures of SES may influence the timing of onset of cognitive impairment. A second limitation comes from the relatively low number of Mexican Americans who exhibited cognitive impairment. Though we detected a statistically significant interaction between education and Hispanic ethnicity, we lack the adequate statistical power to be sure the patterns are true versus an artifact of our particular modeling. As such, our results concerning the differential effects of education between non-Hispanic whites and Mexican Americans should be considered exploratory. Future research using a larger sample of Mexican Americans is needed to better understand the effects of education on cognitive impairment in this population.

### 5.2. Future Directions

Though this study builds on the existing literature on the racial/ethnic differences in the effect of education on cognitive impairment, there are a number of gaps that still need to be addressed. Given the findings of this cross-sectional study that education differentially affects cognitive impairment across racial/ethnic groups, we propose the following future research directions. First, we suggest that a longitudinal study is warranted to examine how education may affect risk of cognitive impairment over time. Moreover, leveraging multiple waves of HRS may allow us to better detect any interaction between education and Mexican ethnicity. Cox hazards models can be estimated to compare risks across race/ethnicity using HRS data, which will allow us to identify differences in age of onset of cognitive impairment. Additionally, future research should examine if other measures of SES such as income and wealth are differentially associated with cognitive impairment across race/ethnicity. Thus, we will be able to identify if cognitive impairment is associated with SES regardless of measurement or if any particular measure has unique effects on cognitive impairment. Future research should build upon the existing study to assess if the relationship between education and cognitive impairment is gendered. For example, regression models stratified by race/ethnicity can include gender and education interaction terms to understand how the impacts of education on cognitive impairment may differ for men and women. Similarly, future research should also examine cohort or generational differences in the relationship between education and cognitive impairment across race/ethnicity as the context of education and therefore its potential influence on health may vary by cohort. Lastly, we suggest a future study using change in cognitive function (instead of cognitive impairment) as the dependent variable. Using measures of adults’ cognitive functioning, we will be able to elucidate how education across race/ethnicity shapes not only adults’ status as being cognitively impaired but also the changes over time in their cognitive abilities. Thus, this research will contribute to the field’s understanding of racial/ethnic disparities in cognitive aging processes as shaped by education.

In summary, we find that blacks and Mexican Americans are at increased risk for cognitive impairment in older adulthood compared to their white peers. While education mediates the cognitive impairment disparity between non-Hispanic whites and Mexican Americans, it does not mediate the disparity between non-Hispanic blacks and non-Hispanic whites. Moreover, we find evidence that education operates differentially by race and ethnicity to affect cognitive impairment such that disparities between racial/ethnic groups examined in this study were widest at lower levels of education and thus consistent with the minority poverty hypothesis. This research contributes to the literature on cognitive functioning disparities by identifying how the effect of education on cognitive impairment is shaped by race and ethnicity. Thus, these findings can inform future research aimed at improving our understanding of the social determinants of cognitive aging outcomes.

## Figures and Tables

**Table 1:** Weighted descriptive statistics (proportions and means) of adults aged 65 and older by race/ethnicity and gender, Health and Retirement Study, 2012.

Focal Variables	Whites		Blacks		Mexican Americans	
	Men (*n =* 3042)	Women (*n =* 4120)	Men (*n =* 464)	Women (*n =* 804)	Men (*n =* 203)	Women (*n =* 270)
Total Cognition	22.03	22.6 *	18.49 *	18.85 *	18.99 *	18.65 *
	(5.02)	(5.37)	(5.44)	(5.83)	(5.06)	(5.86)
% Cognitively Impaired	1.64	1.72	6.74 *	4.78 *	3.35	3.09 **
Age	74.01	75.03 *	72.64 *	73.73 †	72.05 *	72.37 *
	(7.2)	(7.8)	(6.7)	(7.5)	(6.3)	(7.4)
Years of Education	13.51	13.06 *	11.41 *	11.91 *	8.39 *	7.93 *
	(3.3)	(2.9)	(3.2)	(2.9)	(4.6)	(4.5)
Wealth (logged)	5.15	4.98 *	3.90 *	3.20 *†	3.92 *	3.66 *
	(2.1)	(1.9)	(1.2)	(1.5)	(1.1)	(1.1)
Income (logged)	4.71	4.55 *	4.45 *	4.25 *†	4.30 *	4.08 *†
	(1.9)	(2.0)	(2.2)	(2.1)	(1.9)	(2.3)
% Married/Partnered	74.54	48.62 *	56.29 *	27.80 *†	84.29 *	47.23 *†
% Obese	28.82	27.98	31.71	44.33 *†	32.58	37.36
% Never Exercise	17.68	24.77 *	21.45	31.93 *†	21.07	19.42
% Former or Current Smoker	68.09	49.83 *	70.82 *	50.60 *†	74.47	41.59 *†
% Moderate or Heavy Alcohol Drinker	47.08	32.55 *	30.15 *	16.00 *†	39.76 *	10.65 *†
# of Chronic Health Conditions	2.39	2.30 *	2.41	2.67 *†	2.19	2.31
	(1.5)	(1.5)	(1.5)	(1.4)	(1.4)	(1.4)
% Psychiatric Diagnosis	13.94	20.69 *	9.61 *	15.35 †	14.49	22.14 *†
% Depressed	13.73	20.68 *	15.42	24.23 *†	26.53 *	31.91 *

Note: Different from white men ** *p* < 0.01; * *p* < 0.05; standard deviations in parentheses; within race and between gender differences † *p* < 0.05.

**Table 2:** Odds ratios from logistic regression models examining cognitive impairment among U.S. adults, Health and Retirement Study, 2012.

Focal Variables	Model I	Model II	Model III
	OR	OR	OR
Blacks	3.56 ***	2.66 ***	1.60 **
Mexican American	1.96 *	0.63	0.86 **
Years of Education		0.84 ***	0.82 ***
Black x Education			0.81 *
Mexican American x Education			1.15 *
Constant	0.00002	0.000002	0.00002

Note: Whites serve as the reference category. All models adjusted for covariates. * p < 0.05; ** *p* < 0.01; *** *p* < 0.001.
